# Ultra-small low-threshold mid-infrared plasmonic nanowire lasers based on n-doped GaN

**DOI:** 10.1186/s11671-023-03797-6

**Published:** 2023-02-16

**Authors:** Jiahui Zheng, Xin Yan, Xia Zhang, Xiaomin Ren

**Affiliations:** State Key Laboratory of Information Photonics and Optical Communications, Beijing University of Posts and Telecommunications, Beijing, 100876 China

**Keywords:** Nanowire, Laser, Plasmonic, Mid-infrared, n-doped GaN

## Abstract

An ultra-small mid-infrared plasmonic nanowire laser based on n-doped GaN metallic material is proposed and studied by the finite-difference time-domain method. In comparison with the noble metals, nGaN is found to possess superior permittivity characteristics in the mid-infrared range, beneficial for generating low-loss surface plasmon polaritons and achieving strong subwavelength optical confinement. The results show that at a wavelength of 4.2 µm, the penetration depth into the dielectric is substantially decreased from 1384 to 163 nm by replacing Au with nGaN, and the cutoff diameter of nGaN-based laser is as small as 265 nm, only 65% that of the Au-based one. To suppress the relatively large propagation loss induced by nGaN, an nGaN/Au-based laser structure is designed, whose threshold gain has been reduced by nearly half. This work may pave the way for the development of miniaturized low-consumption mid-infrared lasers.

## Introduction

With the increase in global environmental, ecological, and energy issues, the mid-infrared (MIR) wavelength range has attracted unprecedented attention because it contains many basic molecular absorption resonance wavelengths and radiation wavelengths from natural/artificial objects. As the key component of many MIR systems such as gas sensing, free space communications, and surgical operations, MIR laser has always been a key and difficult topic in the field of MIR research [1]. In the past decades, great progress has been made in the realization of miniaturized MIR lasers, including quantum cascade laser (QCL) [2], interband cascade laser (ICL) [3], and vertical-cavity surface-emitting laser (VCSEL) [4]. Typically, the miniaturization of lasers is pursued to achieve a lower lasing threshold [5], higher energy efficiency [6], and higher modulation speed [7]. Possible applications of ultra-small lasers include on-chip optical communication and data processing [8], medical imaging and sensing [9], three-dimensional displays and advanced holography [10], and so on. However, compared with the mature miniaturized lasers in the visible and near-infrared range, the MIR laser possesses a much longer lasing wavelength, which means that a much larger device size is required for optical confinement.

Semiconductor nanowire (NW) is regarded as one of the most promising structures for high-performance miniaturized lasers because of the good stress tolerance [11], bottom-up growth method [12], and intrinsic Fabry–Perot optical cavity [13].Compared to the widely-reported ultraviolet [14], visible [15], and near-infrared [16] NW lasers, only a few MIR NW lasers have been demonstrated in recent years. A 2.6 µm lasing from a single InAs NW was demonstrated by Sumikura et al. of NTT laboratory in 2019 [17]. In 2020, a 3–4 µm lasing was achieved from a single PbS NW fabricated by chemical vapor deposition on Si substrate [18]. Those achievements represent a significant step toward the miniaturization of MIR lasers. However, limited by the optical diffraction, those MIR NW lasers still possess a relatively large diameter of nearly 1 µm. To further decrease the laser size, plasmonic NW laser has been proven to be an effective way [19].

The plasmonic NW laser can realize a subwavelength scale by making use of the surface plasmon polariton (SPP), which is a transverse magnetic-polarized optical surface wave that propagates along the NW–metal interface [20]. Up to date, plasmonic NW lasers have been demonstrated in a variety of NW materials covering the wavelength range from ultraviolet to near-infrared [21]. Among the above lasers, metals such as Au, Ag, and Cu are the most often used for plasmonic metal materials due to their relatively low loss and high conductivity in those wavelength ranges. However, further extension of plasmonic NW lasers to longer wavelengths (such as MIR) encounters severe challenges: the poor spatial confinement of SPPs due to the fundamentally different material optical response of metals in these two wavelength ranges. As a result, the design and realization of ultra-small MIR lasers remain a challenge yet and require thorough studies.

In this paper, a miniaturized MIR plasmonic NW laser, supported by n-doped GaN (nGaN), is proposed and studied. We first discussed the method to select an alternative metallic material and the fundamental MIR plasmonic NW laser structure. We then discussed the characteristic length scales to describe an SPP profile and compared the threshold properties of nGaN-based and Au-based NW lasers. The results show a stronger optical field confinement near the NW–metallic material interface supported by nGaN-based NW laser when compared to Au substrate, leading to a smaller cutoff diameter of NW laser. The next part is focused on decreasing the relatively large propagation loss induced by nGaN. An Au film introduced under the nGaN to form a nGaN/Au hybrid substrate structure can suppress the energy penetration into the metallic material, giving rise to the propagation loss decreasing 2–3 times and the threshold gain decreasing by more than a half. This work provides an effective way for the realization of miniaturized low-consumption MIR lasers and ultra-compact MIR systems.

## Structure and method

### Metallic material selection

**Fig. 1:**
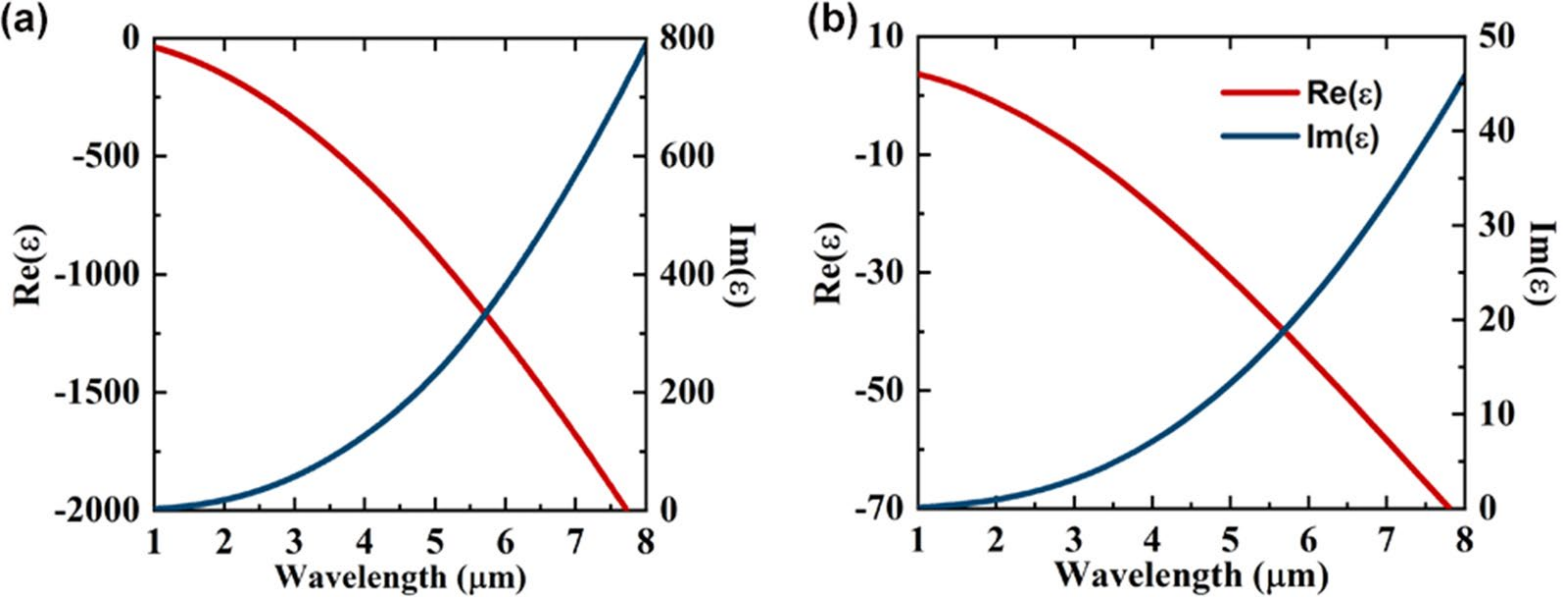
Wavelength-dependent real and imaginary parts of the permittivity of Au **a** and nGaN ($n = 3.7 \times 10^{20} \;{\text{cm}}^{ - 3}$) **b**

Metallic material is one of the essential parts to produce plasmons, mainly including various metals, metal alloys, and heavily doped semiconductors. The value of metallic material’s permittivity plays a vital role in defining whether and how the plasmonic structure supports the plasmonic mode. To generate SPP mode, the real part of the permittivity of metallic material $\varepsilon_{m}^{\prime }$ must be negative, and its magnitude must be greater than that of the gain dielectric material, while the imaginary part of the permittivity $\varepsilon_{m}^{\prime \prime }$ describing the loss is expected to be as small as possible. Traditional SPPs NW usually uses Au as metal materials, and its permittivity often describes by the dataset provided by Palik [22]. However, as shown in Fig. 1a, the absolute value of both $\varepsilon_{m}^{\prime }$ and $\varepsilon_{m}^{\prime \prime }$ of Au increases rapidly with the increase in wavelength. For example, $\left| {\varepsilon_{m}^{\prime } } \right|$ and $\left| {\varepsilon_{m}^{\prime \prime } } \right|$ of Au are 37.94 and 2.3, 655.15 and 144.93, 2124.43 and 232.32 at the wavelength of 1.0, 4.2, and 8 µm, respectively. The large ratio between the metal and NW real permittivity hinders the realization of subwavelength optical confinement, while the large imaginary permittivity leads to huge metal loss, both of which seriously limit the application of Au in MIR SPP laser. Similar issues also exist in other materials such as Ag, Al, transition metal oxides, and nitrides [23, 24].

The Drude model is a simple yet effective model that permits us to predict the permittivity of the plasmonic material as follows [25]:

1 $$\varepsilon_{m} \left( \omega \right) = \varepsilon_{m}^{\prime } (\omega ) + i\varepsilon_{m}^{\prime \prime } (\omega ) = \varepsilon_{\infty } - \frac{{\omega_{p}^{2} }}{{\omega \left( {\omega + i\gamma } \right)}}$$

The plasma frequency $\omega_{p}$ and damping constant $\gamma$ can be described by:

2 $$\omega_{p}^{2} = \frac{{ne^{2} }}{{m^{*} \varepsilon_{0} }}$$

3 $$\gamma = \frac{q}{{\mu m^{*} }}$$

where *n* is the carrier concentration, $\varepsilon_{0}$ and $\varepsilon_{\infty }$ are the free space and the high-frequency permittivity, respectively, $\mu$ is the mobility and $m^{*}$ effective mass.

Through the calculation of the Drude model, it can be found that highly doped III-V semiconductors, such as highly doped InAs and InP, have shown potential in plasmonic applications from long-wave infrared to THZ [26, 27]. However, in the mid-wave infrared range (3–5 μm), most of the doped III-V semiconductors possess a positive real part, unable to generate SPPs. Fortunately, it is found that highly n-doped GaN (nGaN) has a relatively small negative $\varepsilon_{m}^{\prime }$ and a small $\varepsilon_{m}^{\prime \prime }$ in the MIR range, as shown in Fig. 1b. Since different doping methods and concentrations will produce nGaN of different quality, what is also one of the approaches to tune the plasma wavelength. Here, parameters provided by [28] were used: $n = 3.7 \times 10^{20} \;{\text{cm}}^{ - 3}$,$\varepsilon_{\infty } = 5.31$,$\mu = 63\;{\text{cm}}^{2} /Vs$ and $m_{{}}^{*} = 0.202m_{e}$ where $m_{e}$ is the free electron mass. The $\varepsilon_{m}^{\prime }$ and $\varepsilon_{m}^{\prime \prime }$ of nGaN are -8.79 and 3.1, -30.88 and 13.28 at the wavelength of 3.0 and 5.0 µm, respectively, meeting the conditions for generating subwavelength low-loss SPPs.

### Structure and method

**Fig. 2:**
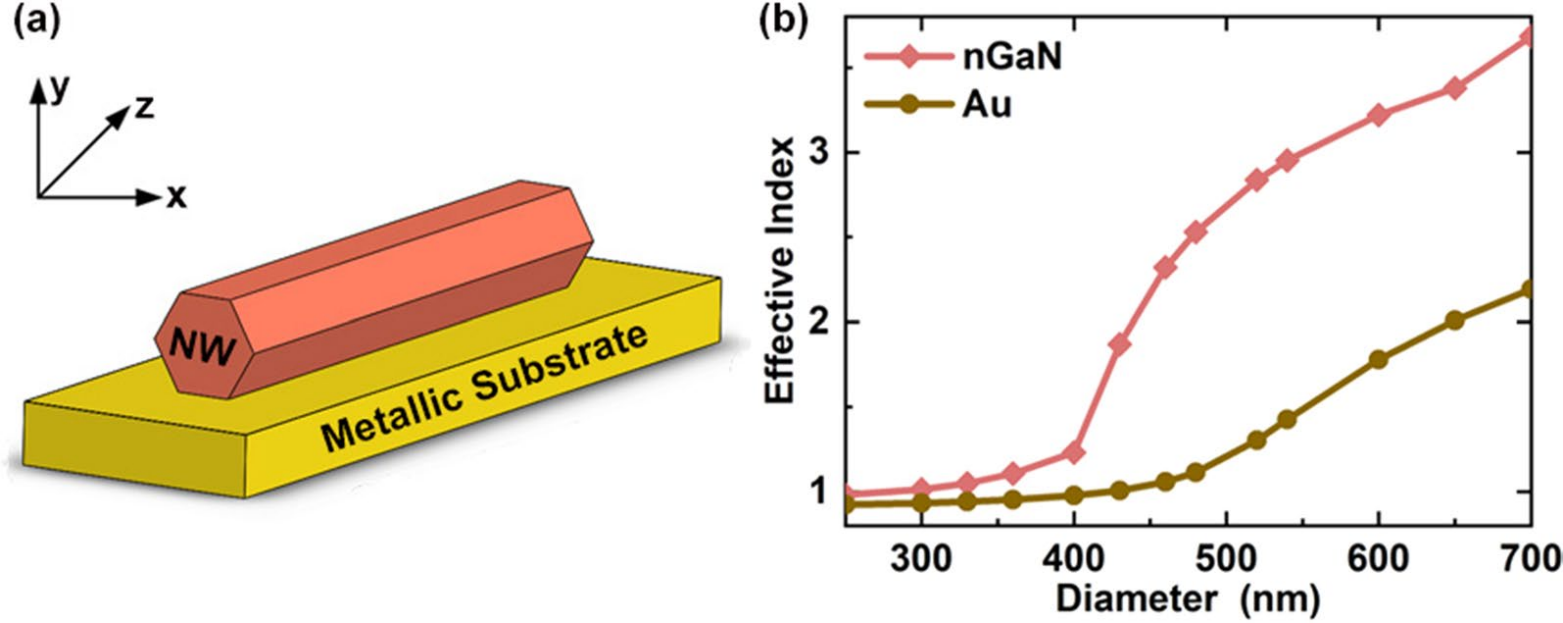
Schematic structure of plasmonic NW laser **a** and mode effective index of plasmonic NW lasers **b**

Figure 2a shows the typical schematic structures of plasmonic NW lasers studied in this work. A single semiconductor NW with cleaved ends defining a Fabry–Perot cavity lies on a metal substrate. The NW has a hexagonal cross section, and only one side facet is in contact with the substrate. The length of NW is set to 14 μm [17]. In the following discussions, the simulated wavelength of 4.2 µm was focused, corresponding to the optical probe for carbon dioxide [29, 30]. The material of NW is monocrystalline InAs_0.9_Sb_0.1_ to achieve lasing at 4.2 µm [31]. At this wavelength, $\left| {\varepsilon_{m}^{\prime } } \right|$ of InAsSb is 12.24, and the $\varepsilon_{m}^{\prime }$ and $\varepsilon_{m}^{\prime \prime }$ of nGaN are − 21.15 and 8.15, respectively.

Lumerical FDTD Solutions is used to simulate the electromagnetic field distribution in a plasmonic NW laser. The stretched coordinate perfectly matched layer absorbing boundary condition is adopted to minimize the reflections in the background. The mode source of 4.2 µm free space wavelength inside the NW can then excite a single SPP mode which propagates from the launch point toward the end of the NW. 3D frequency-domain profile and power monitors are utilized to obtain the electromagnetic field components, and the mode monitor is used to calculate the end facet reflectivity. The mesh step is set to as small as 5 nm in the z-direction for precise analysis. Figure 2b presents the effective index for supported guiding SPP mode versus NW diameter at a wavelength of 4.2 µm. The mode effective index increases with the diameter, and the effective index of nGaN-based NW lasers is always larger than that of Au-based lasers at the same diameter.

## Results and discussion

### SPP profile penetration depth

**Fig. 3:**
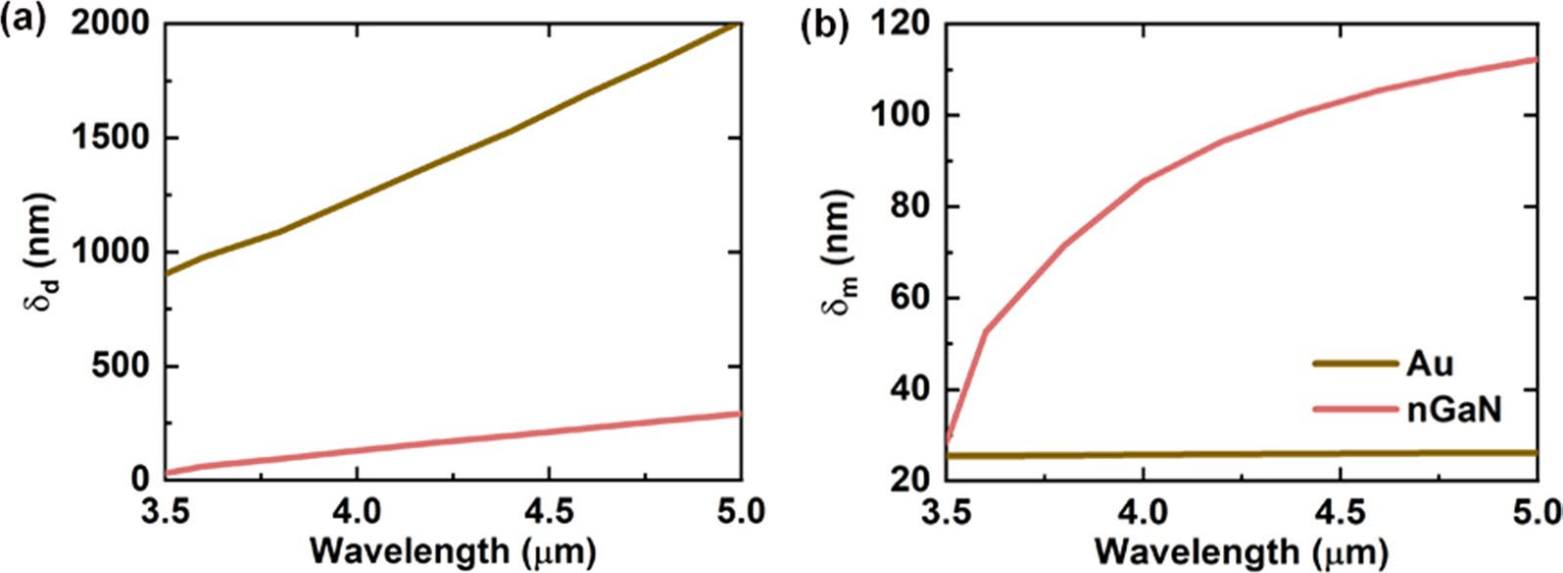
Wavelength-dependent penetration depth into dielectric **a** and metallic **b** layer of SPPs on Au and nGaN substrates

An SPP is a couple exciton comprising a charge density wave in metallic material and an electromagnetic field that peaks at the metallic–dielectric material interface and decays exponentially into both media. To better understand the SPP profile in plasmonic NW lasers, we first characterize SPPs at the interface of metallic-InAsSb layers by their penetration depths that can be described by $\varepsilon_{m}^{\prime }$ and $\varepsilon_{m}^{\prime \prime }$ [32]:

4 $$\delta_{d} = \frac{{\lambda_{0} }}{2\pi }\left| {\frac{{\varepsilon_{d}^{\prime } + \varepsilon_{m}^{\prime } }}{{\left( {\varepsilon_{d}^{\prime } } \right)^{2} }}} \right|^{\frac{1}{2}}$$

5 $$\delta_{m} = \frac{{\lambda_{0} }}{2\pi }\left| {\frac{{\varepsilon_{d}^{\prime } + \varepsilon_{m}^{\prime } }}{{\left( {\varepsilon_{m}^{\prime } } \right)^{2} }}} \right|^{\frac{1}{2}}$$

where the $\delta_{d}$ and $\delta_{m}$ are the penetration depth into the dielectric and metallic layer, $\varepsilon_{d}$ and $\varepsilon_{m}^{{}}$ are the permittivity of NW and metallic material as a function of wavelength, and $\lambda_{0}$ is the incoming radiation wavelength. Apparently, the total penetration depth $\left( {\delta_{d} + \delta_{m} } \right)$ determining the compactness of the mode only depends on the real part of the permittivity, giving a measure of the minimum thickness required for the dielectric and metallic layers. On the one hand, smaller $\delta_{d}$ means a possible small size device, also smaller mode overlaps with the gain medium. It can be seen from Fig. 3a that for the wavelength in MIR spectral, the $\delta_{d}$ of Au-based SPP is far greater than that of nGaN-based SPP. For example, the $\delta_{d}$ into Au is 1384 nm at 4.2 µm wavelength, while that into nGaN is 163 nm. As the wavelength increases, this phenomenon becomes more apparent, limiting the applications of metals in ultra-small MIR SPPs. On the other hand, the $\delta_{m}$ into Au is much smaller than that into nGaN at the same wavelength. As shown in Fig. 3b, $\delta_{m}$ into Au is maintained at a small value of 26 nm nearly independent of the wavelength. The $\delta_{m}$ into nGaN is 94 nm at the 4.2 µm wavelength, approximately 3.6 times that into Au. Though the $\varepsilon_{m}^{^{\prime\prime}}$ of nGaN is much smaller than that of Au, the $\delta_{m}$ brings another degree of freedom to metal propagation loss, which will be described in detail in the next section.

In summary, the total penetration depth of nGaN is much smaller than that of Au in MIR wavelength. The conventional SPPs here in Au are more like surface-current guided modes moving away from the Au–InAsSb interface, which is unable to provide strong confinement of the electromagnetic field. Instead, nGaN can provide a more tightly bound SPP mode in MIR, allowing for the realization of ultra-small plasmonic NW lasers.

**Fig. 4:**
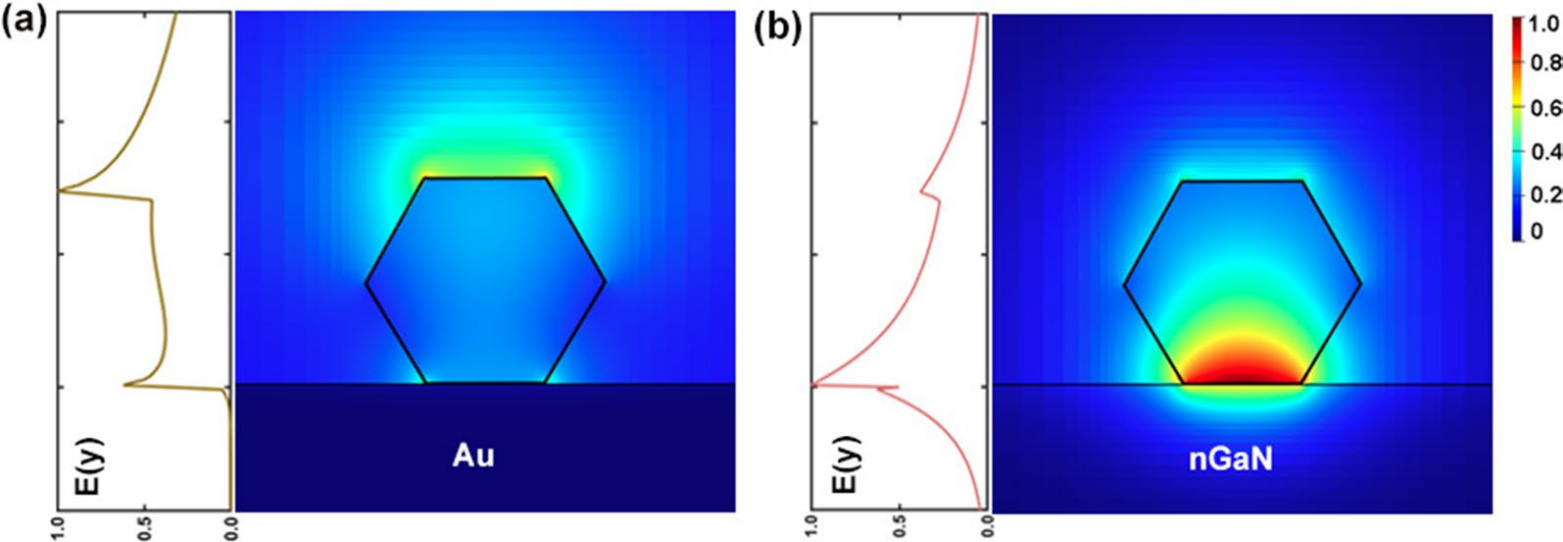
The transverse modal profiles of plasmonic NW laser on Au **a** and nGaN substrate **b** (where the NW diameter = 500 nm). The inset on the left shows the normalized electric field distribution along Y-axis

In addition, the transverse electric field distribution for InAsSb NW on Au and nGaN substrate with a diameter of 500 nm at the 4.2 µm wavelength is presented in Fig. 4a and b, respectively. All the electric field intensity is normalized by the maximum electric field intensity obtained in the structures and can be directly reflected by color. It can be seen that although the refractive index difference between the NW and air further restricts the light field in the dielectric, a large part of energy still leaks outside the NW on Au, forming a leaky mode at the NW–air interface. In contrast, most of the energy is strongly confined in a small area near the NW–nGaN interface for NW on nGaN. The normalized electric field intensity along the y-axis on the left side of the figures can help us intuitively grasp the compactness of SPP modes.

### Threshold properties of nGaN-based NW laser

**Fig. 5:**
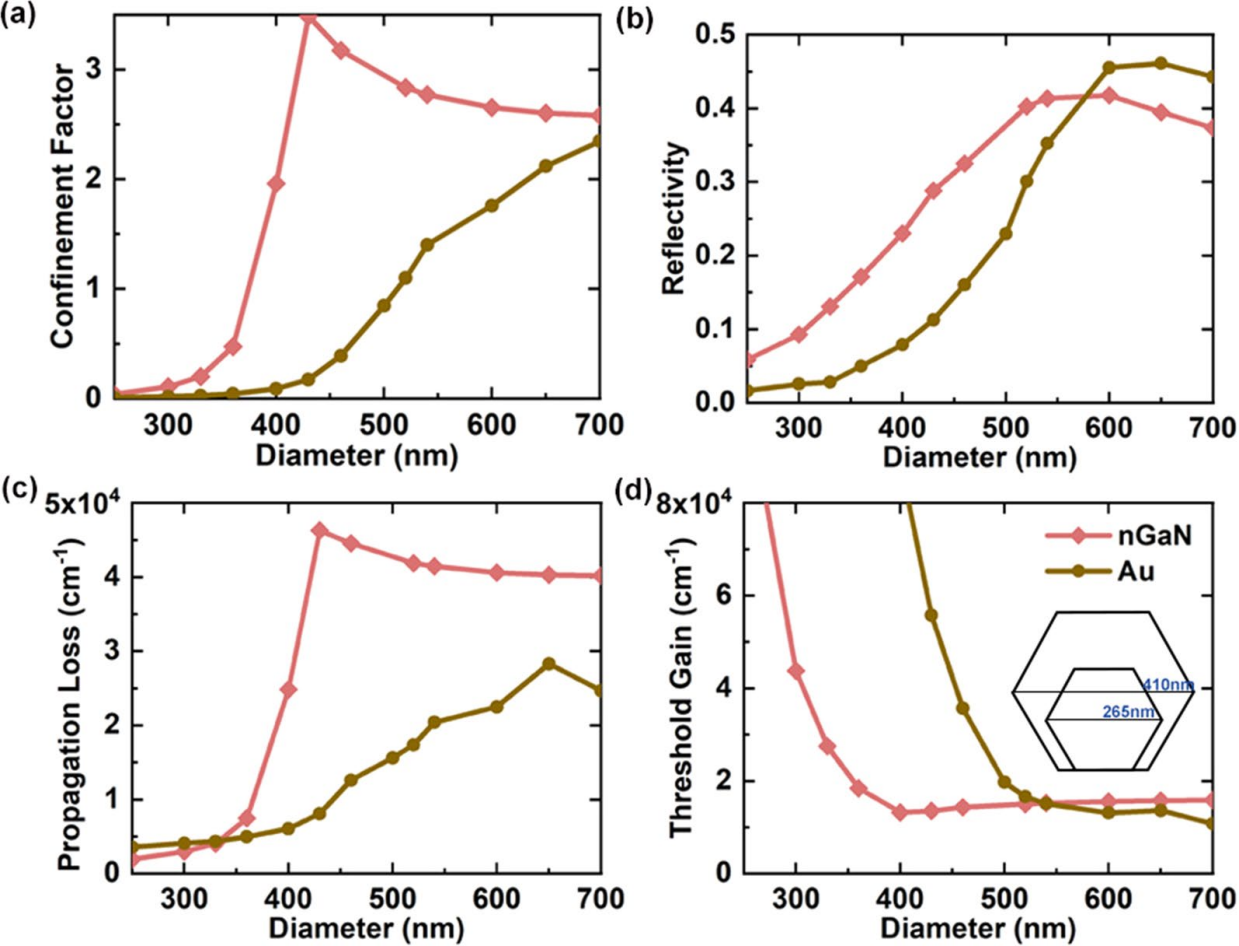
Diameter-dependent mode confinement factor (CF) **a**, end facet reflectivity **b**, propagation loss **c,** and threshold gain **d** for Au-based (gold lines) and nGaN-based (red lines) NW lasers. The inset in **d** provided a physical intuition for the cutoff diameter’s difference

To investigate the threshold properties of the proposed NW lasers, the confinement factor (CF), end facet reflectivity(R), propagation loss $\alpha_{p}$, and threshold gain $g_{th}$ are calculated, as presented in Fig. 5.

The CF for a NW laser can be calculated as follows [33]:

6 $$\Gamma_{s} = \frac{{{c \mathord{\left/ {\vphantom {c {\nu_{g} }}} \right. \kern-0pt} {\nu_{g} }}}}{{n_{s} }}\frac{{\iint\limits_{s} {{\text{Re}} \left[ {\frac{d(\varepsilon \omega )}{{d\omega }}\left| E \right|^{2} } \right]dxdy}}}{{\frac{1}{2}\iint\limits_{\infty } {\left\{ {{\text{Re}} \left[ {\frac{d(\varepsilon \omega )}{{d\omega }}\left| E \right|^{2} + \mu \left| H \right|^{2} } \right]} \right\}dxdy}}}$$

7 $$v_{g} = \frac{{\frac{1}{2}\iint\limits_{\infty } {\left( {E \times H^{*} } \right)dxdy}}}{{\frac{1}{4}\iint\limits_{\infty } {\left\{ {{\text{Re}} \left[ {\frac{d(\varepsilon \omega )}{{d\omega }}\left| E \right|^{2} + \mu \left| H \right|^{2} } \right]} \right\}dxdy}}}$$

where E and H are the complex electric and magnetic fields of the lasing modes, respectively, $\nu_{g}$ is the average energy velocity, and n_s_ is the refractive index of NW. The permittivity $\varepsilon$ here is replaced by $d\left( {\varepsilon \omega } \right)/d\omega$ in view of material dispersion, and $\mu$ is the vacuum permeability. As shown in Fig. 5a, the CF of nGaN-based lasers is higher than that of Au-based lasers, which is attributed to the stronger bound SPP mode and slower energy velocity. For example, at a diameter of 400 nm, the CF of nGaN-based NW laser is 1.96, which is 22.1 times that of an Au-based NW laser. The larger CF means a better energy exchange of the optical field and gain medium, which is one of the prerequisites for a low lasing threshold.

The R of the NW laser strongly depends on the mode type, lasing frequency, and the radius of the NW and can be calculated as the ratio of the intensity of the backward to the forward wave[34]. Figure 5b shows that the R of the nGaN-based NW laser is larger than that of the Au-based NW laser at small diameters (< 550 nm). Since the NW laser works at the same single wavelength, the larger R is mainly due to the better light field confinement at the same diameter. The mirror loss of a Fabry–Perot cavity can be calculated as:

8 $$\alpha_{m} = \frac{1}{2L}\ln \frac{1}{{R^{2} }}$$

where L is the length of the NW. Owing to the logarithmic dependence of mirror loss and reflectivity, the value of $\alpha_{m}$ is very sensitive to the value of R at the same NW length, especially at low reflectivity.

The propagation loss caused by the metallic material is one of the major factors limiting the performance of plasmonic NW lasers. The propagation loss per unit length can be calculated by [35]:

9 $$\alpha_{p} = \frac{{\omega \varepsilon_{0} \iint\limits_{m} {\varepsilon_{m}^{\prime \prime } \left| E \right|^{2} dxdy}}}{{\iint\limits_{\infty } {\left( {E \times H^{*} } \right)dxdy}}}$$

Figure 5c shows the diameter-dependent $\alpha_{p}$ of NW lasers based on nGaN and Au. Notably, the nGaN-based NW laser still exhibits a larger propagation loss than that of the Au-based laser for all diameters, even though the $\varepsilon_{m}^{^{\prime}}$ of nGaN is only 3.2% that of Au. This is because the $\alpha_{p}$ of lasers depends not only on the $\varepsilon_{m}^{^{\prime\prime}}$, but also on the integration of light field energy as described in Eq. (9). When the diameter is smaller than 550 nm, the $\alpha_{p}$ of the Au-based laser drops rapidly to near zero as the NW diameter decreases, indicating that there is almost no light field in Au layer. The $\alpha_{p}$ of nGaN-based lasers maintains a relatively stable value when the NW diameter is greater than 430 nm. When the diameter is smaller than 430 nm, $\alpha_{p}$ also decreases with the decrease in NW diameter.

The threshold gain $g_{th}$ is the minimum gain compensation required for lasing and can be calculated by [36]:

9 $$g_{th} = \frac{1}{\Gamma }(\alpha_{m} + \alpha_{p} )$$

It is clear from Fig. 4d that the two curves have an intersection around the 520 nm diameter. For diameter smaller than 550 nm, the nGaN-based NW laser exhibits a lower $g_{th}$ than that of the Au-based one. For example, at a diameter of 400 nm, the $g_{th}$ of the nGaN-based NW laser is only 14.7% that of the Au-based one. Moreover, the cutoff diameter of the nGaN-based NW laser is as small as 265 nm, only 65% of that of the Au-based one. The above results demonstrate the feasibility of miniaturized nGaN-based NW lasers in the MIR wavelength range.

### Threshold properties of nGaN/Au-based NW laser

**Fig. 6:**
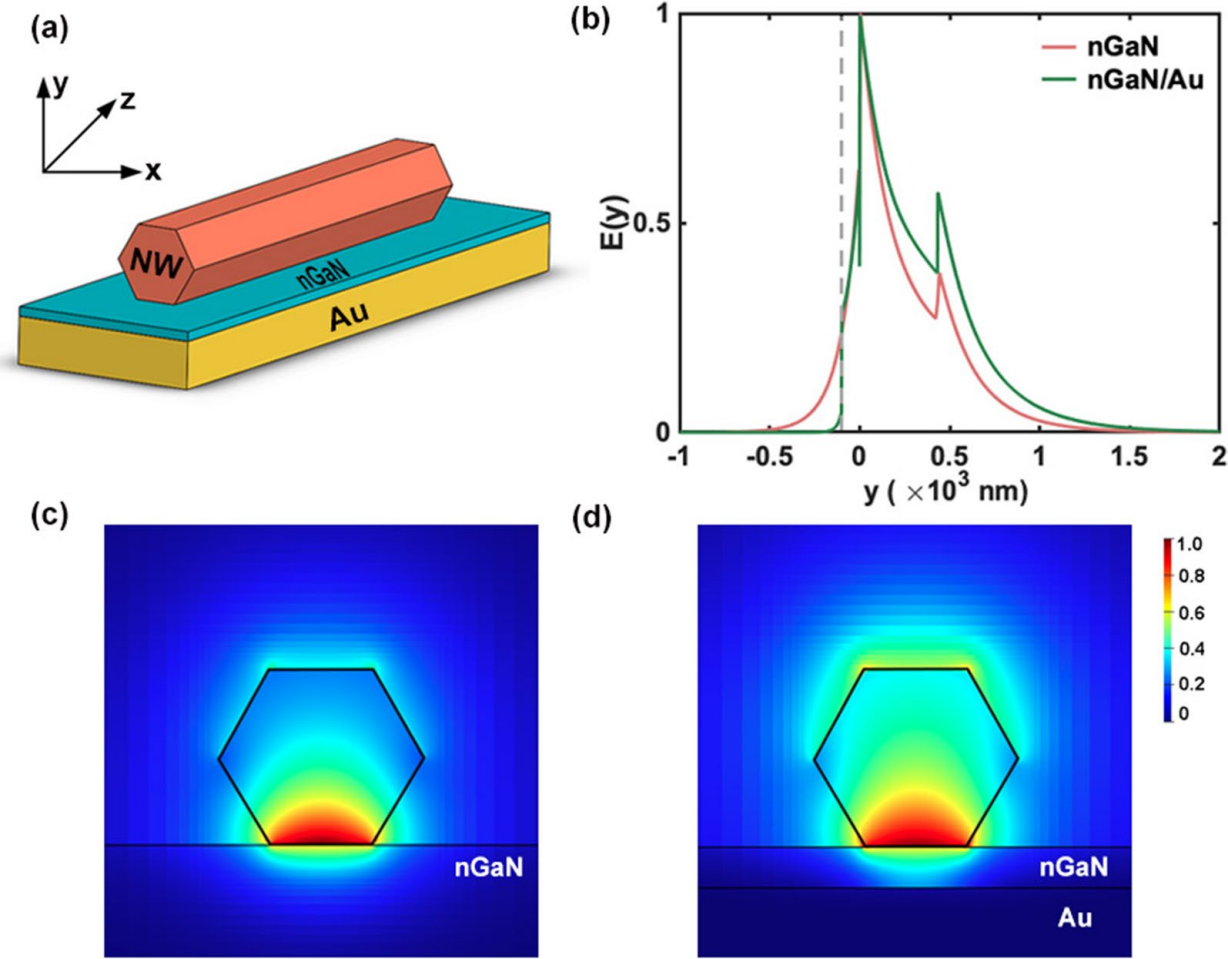
**a** Schematic structure of the nGaN/Au-based NW laser. **b** Electric field magnitude distribution of nGaN and nGaN/Au-based NW lasers along the direction perpendicular to the substrate surface. The transverse electric field distributions of these plasmonic NW lasers are **c** and **d**, respectively

As discussed above, although the nGaN layer significantly enhances the mode confinement at small diameters, the large penetration depth into the nGaN layer results in a large propagation loss, adding some difficulties to lasing. To solve this problem, an nGaN/Au hybrid substrate structure is proposed, as shown in Fig. 6a. The growth of thick GaN films on Au/SiO2 substrates by chemical vapor deposition has been reported in [37]. In this structure, the 100-nm-thick nGaN layer is used to generate strongly confinement SPPs, while the Au substrate is used to suppress the penetration of light field into the metallic material.

Figure 6b shows the normalized electric field distribution along the direction perpendicular to the substrate surface for nGaN- and nGaN/Au-based NW lasers. For the nGaN-based NW laser, the electric field decays slowly in the nGaN layer and finally decays to zero at -500 nm. After the introduction of the Au layer, the electric field drastically decays from -80 nm and decays to zero at -150 nm. Figure 6c and d shows that the field component of the nGaN/Au-based laser is reduced in the substrate, while is significantly increased in the NW.

**Fig. 7:**
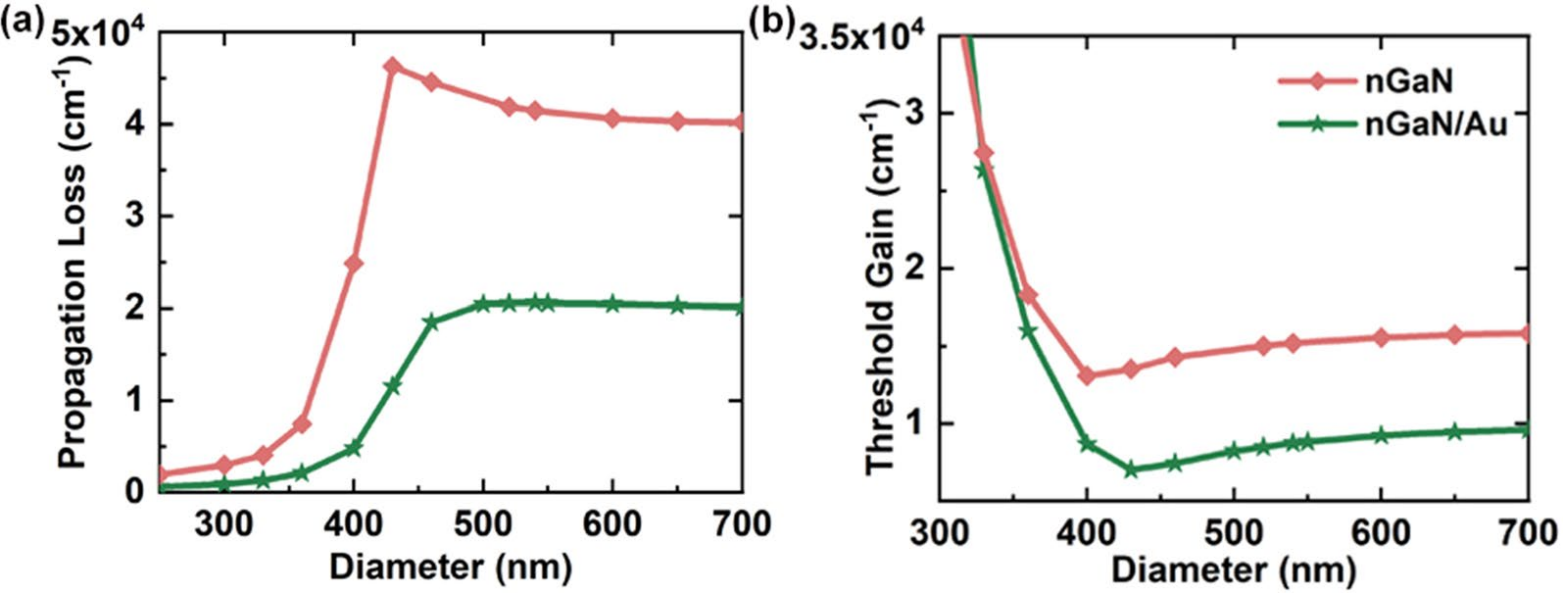
Propagation loss **a** and threshold gain **b** versus NW diameter for the plasmonic laser on nGaN (red lines) and nGaN/Au (green lines) substrate

Figure 7 shows the diameter-dependent propagation loss and threshold gain properties of nGaN- and nGaN/Au-based NW lasers. The propagation loss has decreased 2 ~ 3 times by using the nGaN/Au hybrid substrate, as shown in Fig. 7a. For example, at a diameter of 460 nm, the $\alpha_{p}$ of nGaN/Au-based NW laser is 14297.7 cm^−1^, only 32.1% that of the nGaN-based one. The small $\alpha_{p}$ results in a low threshold gain, as shown in Fig. 7b. Over a broad diameter range of 350–700 nm, the nGaN/Au-based NW laser exhibits a much lower threshold compared to the nGaN-based one. The lowest threshold gain for the nGaN/Au-based NW laser is 7027.8 cm^−1^ obtained at 430 nm, only 52% of that of the nGaN-based NW laser.

## Conclusion

In summary, MIR plasmonic InAsSb NW lasers supported by n-doped GaN have been proposed and studied. The results show that the penetration depth into the dielectric is substantially decreased by replacing Au with nGaN, and the cutoff diameter is as small as 265 nm, only 65% of that of the Au-based one. As the nGaN film generates a relatively large propagation loss, an nGaN/Au hybrid substrate structure is proposed to suppress the energy penetration into the metallic material. Compared with the nGaN-based NW, the propagation loss has been reduced by 2 ~ 3 times, and the threshold gain has been reduced by nearly half. The structures show great potential in ultra-small low-consumption MIR devices and related microsystems. Moreover, by simply tuning the doping level, nGaN is expected to support MIR SPPs with different wavelengths for various applications.

## Data Availability

The datasets used and/or analyzed during the current study are available from the corresponding author upon reasonable request.

## Abbreviations

MIR: Mid-infrared
QCL: Quantum cascade laser
ICL: Interband cascade laser
VCSEL: Vertical surface-emitting laser
NW: Nanowire
SPP: Surface plasmon polariton
nGaN: N-doped GaN
CF: Confinement factor
R: Reflectivity
